# Anatomical localization of the emergence point of the accessory zygomaticotemporal nerve using the marginal tubercle for its clinical implications

**DOI:** 10.1038/s41598-026-56624-0

**Published:** 2026-06-05

**Authors:** Shin Hyo Lee, Hyun Jin Shin, You-Jin Choi, Hee-Jin Kim, Kang-Jae Shin

**Affiliations:** 1 Department of Anatomy, Wonkwang University School of Medicine, Iksan, Republic of Korea; 2 Jesaeng-Euise Clinical Anatomy Center, Wonkwang University School of Medicine, Iksan, Republic of Korea; 3 Department of Ophthalmology, Konkuk University Medical Center, Seoul, Republic of Korea; 4 Research Institute of Medical Science, Konkuk University School of Medicine, Seoul, Republic of Korea; 5 Department of Anatomy, College of Medicine, Konkuk University, Chungju, Republic of Korea; 6 Division in Anatomy and Developmental Biology, Department of Oral Biology, Yonsei University College of Dentistry, Seoul, Republic of Korea; 7 Department of Anatomy and Cell Biology, Dong-A University College of Medicine, Busan, Republic of Korea

**Keywords:** Zygomaticotemporal nerve, Accessory branch, Marginal tubercle, Lateral canthus, Deep temporal fascia, Nerve block, Anatomy, Diseases, Health care, Medical research

## Abstract

Zygomaticotemporal nerve (ZTN) blocks are widely used for temporal procedures, yet incomplete anesthesia may occur when accessory ZTN branches (aZTN) are not accounted for. This study aimed to localize the emergence point of the aZTN (aEP) using the marginal tubercle (MT) as a palpable landmark and to provide in vivo ultrasound depth information relevant to safe, accurate needle placement. We dissected 36 hemifaces from 20, embalmed cadavers to identify the aZTN and measure the aEP relative to the MT and the lateral canthus (LC). In vivo ultrasonography was performed in 23 healthy volunteers to measure the depth from the skin surface to the superficial layer of the deep temporal fascia (sDTF) near the MT. The aZTN was identified in 7 of 36 hemifaces (19.4%), with the aEP clustering within a few millimeters of the MT and showing three vertical patterns (superior, same level, or inferior). Using the LC as a reference, the aEP was located 22.3 ± 2.4 mm lateral and 5.8 ± 2.7 mm superior to the LC. Ultrasonography showed a mean skin-to-sDTF depth of 5.92 ± 0.99 mm. Two specimens demonstrated a bone-emergent aZTN variant (mean MT–bony emergence distance, 4.2 ± 0.1 mm). In conclusion, MT- (and LC-) referenced mapping, combined with ultrasound-derived depth to the sDTF, provides practical anatomical guidance for localizing the aEP and may help refine future MT-centered approaches for addressing accessory ZTN pathways.

## Introduction

The zygomaticotemporal nerve (ZTN), a branch of the maxillary division of the trigeminal nerve, provides sensory innervation to the lateral forehead and anterior temporal region^1^. Because of its sensory distribution, ZTN blocks have been widely utilized in diverse craniofacial procedures for managing temporal headaches, neuralgia, and postoperative pain, as well as for achieving anesthesia during aesthetic interventions in the temporal area^2,3^. They are also applied in aesthetic procedures such as temporal lifts and filler injections^4,5^. Despite their clinical value, ZTN blocks may sometimes have reduced efficacy even with conventional injection technique, which might be attributed to the presence of potential accessory branches of the ZTN (aZTN)^6,7^. Comparable challenges have been noted in other craniofacial nerves. In a cadaveric study, Shin et al.^8^ identified accessory infraorbital nerves in 18.2% of 44 cases, a finding that underscores the potential for anesthesia failure during midface surgery when blocks are placed at conventional anatomical sites.

Previous studies have highlighted variations of ZTN, including reports of presence of the aZTN. Grimes et al.^9^ documents the presence of multiple zygomaticotemporal foramina, with studies identifying two ZTFs in 5 out of 8 subjects. Totonchi et al.^7^ found that at least one additional accessory branch in 60% of patients and 15% had two accessory branches. Micro-CT studies further demonstrate the complexity of the ZTN’s course, revealing that zygomaticotemporal canals (ZTCs) can branch from the intrabony canal of the zygomaticofacial canal in 71.4% of cases^10^. Given that accessory ZTN branches can follow independent anatomical pathways distinct from the main ZTN trunk, research clarifying the existence and precise anatomical knowledge of these accessory branches is essential to enhance the success rate of regional blocks of temporal area^7,11^. However, existing research, while thoroughly documenting these anatomical variations and the presence of accessory branches, often focuses on merely reporting these anatomical discrepancies without providing explicit guidance on how to effectively block these newly identified or variable accessory branches for successful intervention. This knowledge gap contributes to challenges in clinical practice, such as the reported high block failure rates associated with traditional landmark-based techniques^12,13^.

Previous research by Choi and Kim^11^ demonstrated that the marginal tubercle (MT)—a small bony prominence located along the lateral border of the frontal process of the zygomatic bone—serves as a clinically useful and reliable external landmark for identifying the emergence point of the ZTN. Considering that aZTN may follow independent courses from the main trunk and contribute to incomplete anesthesia or treatment failure, further clarification of their precise emergence points is warranted. Since the main ZTN branch has been successfully localized using the MT as a reference point, applying the same landmark-based approach to aZTN would provide clinicians with a unified and practical framework for performing nerve blocks. Therefore, this study aimed to determine the precise emergence points of the aZTN (aEP) using the MT as a consistent and clinically accessible landmark. Additionally, in vivo ultrasonography was employed to measure the depth from the skin surface to the aEP, providing clinically relevant information for safe and accurate needle placement during nerve block procedures.

## Methods

All procedures were conducted in accordance with the Declaration of Helsinki of the World Medical Association. Ethical approval was obtained from the institutional review boards of Dong-A University College of Medicine (No. 2-1040709-AB-N-01-202404-BR-003-02) and Yonsei University College of Dentistry (No. 2-2017-0023). For the cadaveric component, 20 legally donated, embalmed Korean cadavers were dissected to investigate the anatomy of the temporal region. The final sample comprised 36 hemifaces (15 males and 5 females; mean age at death, 83 years; range, 67–93 years), including 21 right and 15 left hemifaces. Four hemifaces were excluded due to anatomical disruption. None of the included specimens showed gross pathology or signs of previous surgical intervention in the examined areas.

### Cadaveric dissection and measurements

Anatomical dissections were performed using a transcutaneous layer-by-layer approach. The aZTN was defined as an additional sensory branch distinct from the main ZTN. A branch was identified as an aZTN when it could be traced separately from the main ZTN and showed at least one of the following anatomical features: an independent course, bony emergence point, a smaller caliber than the main ZTN, or an emergence location different from the expected main ZTN emergence point near the marginal tubercle. Thus, the term “accessory” was used not simply to indicate small size, but to describe a branch with an anatomically separate pathway or emergence pattern from the main ZTN.

**Fig. 1:**
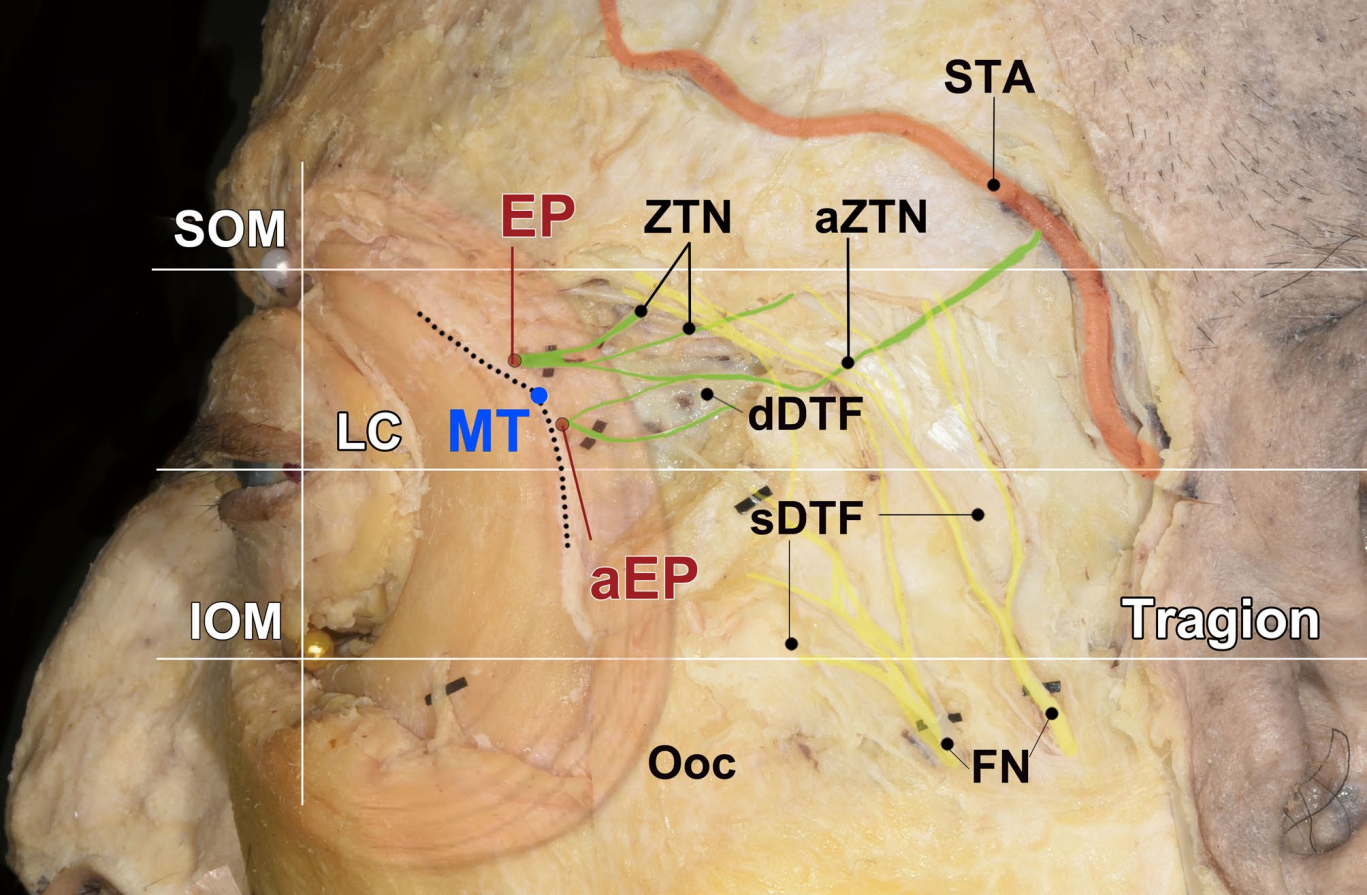
Accessory zygomaticotemporal nerve in relation to the marginal tubercle. Cadaveric dissection of the temporal region showing the zygomaticotemporal nerve (ZTN) and an accessory zygomaticotemporal nerve branch (aZTN). The emergence point of the main ZTN is designated as the EP, whereas the emergence point of the accessory branch is designated as the accessory emergence point (aEP). The aEP is defined as the site where the aZTN penetrates the deep layer of the deep temporal fascia (dDTF). The marginal tubercle (MT) is used as a bony landmark, with reference axes drawn through the lateral canthus (LC) to define spatial relationships. Key surrounding structures, including the superficial temporal artery (STA), facial nerve branches (FN), and fascial layers, including the superficial and deep layers of the deep temporal fascia (sDTF and dDTF), are shown.

The aEP was defined as the site where the aZTN penetrates the deep layer of the deep temporal fascia (dDTF) and emerges superficially to contact the lateral border of the frontal process of the zygomatic bone (Fig. 1). The aZTN are characterized by their deviation from this typical path of the main ZTN, often being smaller in size or emerging from non-typical locations or with distinct, independent pathways. Based on the vertical relationship between the aEP and the MT, accessory emergence patterns were classified into three types: Type 1 (aEP located superior to the MT), Type 2 (aEP at the same horizontal level as the MT), and Type 3 (aEP located inferior to the MT). For each type, linear distance from the MT and the depth from the skin surface to the superficial layer of the deep temporal fascia (sDTF) were recorded.

The aZTN was traced along its course originating from the aEP. The position of the aEP was determined relative to two anatomical landmarks (Fig. 1): (1) the MT, defined as the most prominent point along the lateral border of the frontal process of the zygomatic bone^11^, and (2) the lateral canthus (LC), corresponding to the junction of the upper and lower eyelids. The primary outcome was the distance from the MT to the aEP, and the secondary outcome was the distance from the LC to the aEP. For reference, general facial dimensions were also recorded, including the horizontal distance from the LC to the tragion and from the medial orbital margin (MOM) to the lateral orbital margin (LOM), and the vertical distance from the superior orbital margin (SOM) to the inferior orbital margin (IOM).

To summarize, we measured the following parameters (referred to as #1 to #7) on cadavers using a scale bar at a precision of 0.1 mm:

1. Linear distance from the MT to the aEP.
2. Horizontal distance from the LC to the aEP.
3. Vertical distance from the LC to the aEP.
4. Horizontal distance from the LC to the MT.
5. Vertical distance from the LC to the MT.
6. Horizontal facial dimension from the LC to the tragion.
7. Horizontal distance from MOM to LOM.
8. Vertical facial dimension from the SOM to the IOM.

### Ultrasonography depth measurement

In the temporal region, the superficial temporal fascia (STF), also known as the temporoparietal fascia and continuous with the superficial musculoaponeurotic system (SMAS), lies superficially. Deeper to this layer, the deep temporal fascia (DTF) divides around the superficial temporal fat pad into superficial and deep layers, referred to here as the sDTF and dDTF, respectively. The ZTN and aZTN course within the deeper fascial plane between the sDTF and dDTF, while the frontal branch of the facial nerve lies more superficially, between the STF and sDTF^14^.

In vivo ultrasonography was performed in 23 healthy volunteers with no facial anomalies (11 males and 12 females; mean age, 25.4 ± 3.0 years; range, 21–34 years). Exclusion criteria for the ultrasonographic examination included a history of facial trauma, facial or temporal surgery, congenital craniofacial anomaly, known soft-tissue mass or inflammation in the temporal region, prior filler or botulinum toxin injection in the temporal area, and any condition that could distort the normal fascial anatomy. Written informed consent was obtained from all participants before examination. Left sides of the face were evaluated All sonographic examinations were performed using a real-time ultrasound scanner (E-CUBE 15 EX, ALPINION, Seoul, Korea) with a 30 mm-wide linear-array transducer (8.0–17.0 MHz; IO8-17 High-Frequency Hockey Stick, Seoul, Korea). Participants were examined in a sitting position, approximating the natural posture, to minimize the gravitational effects on soft tissues. Sagittal sonograms of the temporal region were obtained near the MT.

Ultrasonography was used to measure the depth from the skin surface to the sDTF. The aZTN itself was not directly visualized by ultrasound. Rather, the skin-to-sDTF depth was measured as an in vivo reference for the target interfascial plane, where the ZTN and, in its usual course, the aZTN are expected to course between the sDTF and the dDTF based on cadaveric anatomy^11^.

### Statistical analyses

Statistical analyses (IBM SPSS Statistics software. version 27.0, IBM Corporation, Armonk, NY, USA) were primarily descriptive because the key anatomical findings were based on the subset of aZTN-positive hemifaces. Continuous variables are presented as mean ± standard deviation. Normality was assessed using the Shapiro–Wilk test; however, given the small number of aZTN-positive cases, inferential analyses involving aEP location were considered exploratory. Sex- and laterality-based comparisons were performed for descriptive purposes. Pearson’s correlation coefficients were calculated only to explore potential associations between facial dimensions and LC-based aEP distances, and these results were interpreted as hypothesis-generating rather than confirmatory. *P* values, when reported, were interpreted descriptively, and no definitive statistical significance was inferred from these exploratory analyses.

## Results

**Fig. 2:**
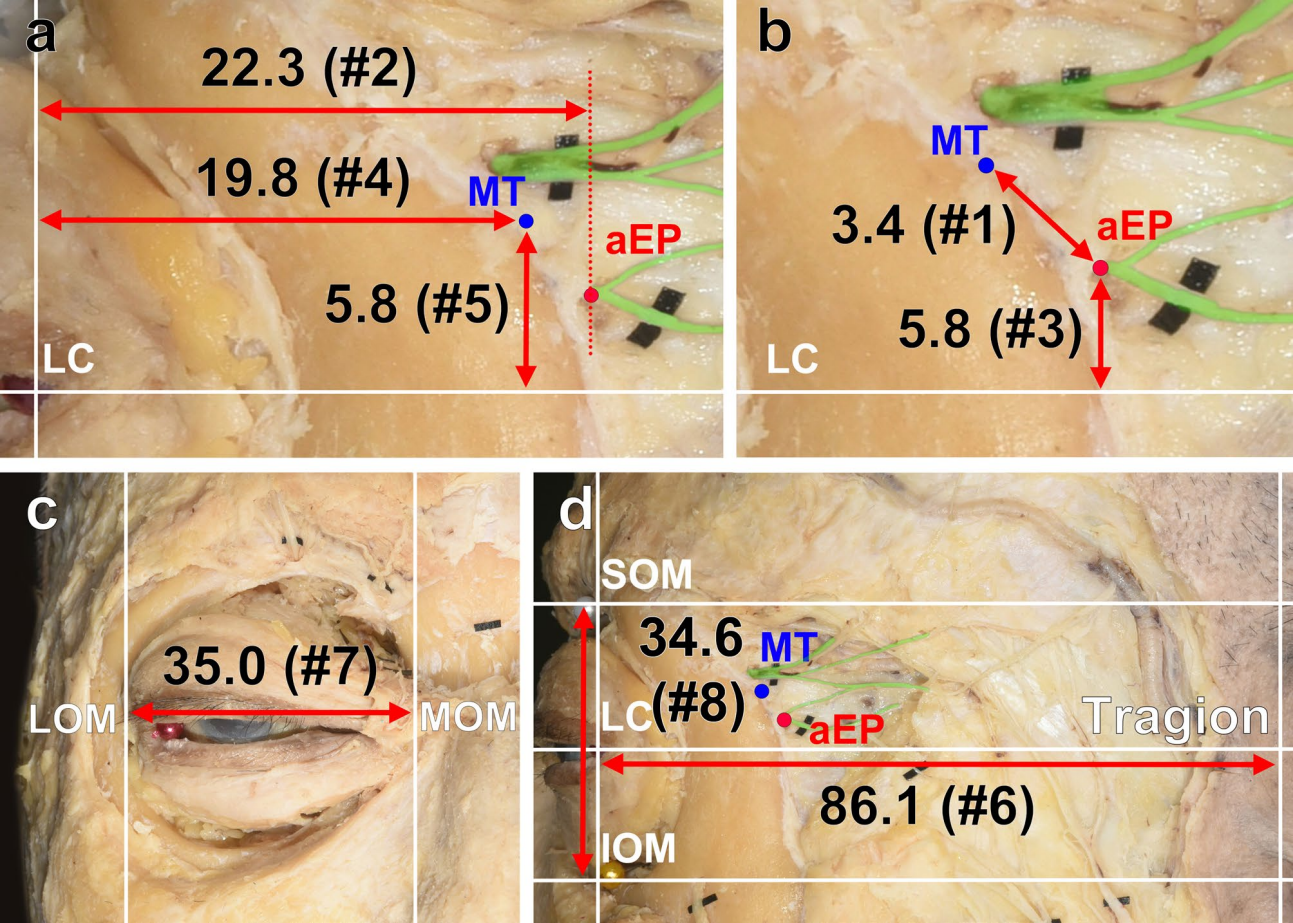
Measurement of the accessory emergence point relative to facial landmarks. Cadaveric measurements of the accessory emergence point (aEP) relative to the marginal tubercle (MT) and lateral canthus (LC). (**a**) Horizontal distances from the LC to the aEP (#2) and to the MT (#4), and vertical distance from the LC to the MT (#5). (**b**) Linear distance from the MT to the aEP (#1) and vertical distance from the LC to the aEP (#3). (**c**) Horizontal orbital width from the medial to lateral orbital margin (MOM–LOM, #7). (**d**) Vertical orbital height from the superior to inferior orbital margin (SOM–IOM, #8) and horizontal facial width from the LC to the tragion (#6). All values are in millimeters.

**Fig. 3:**
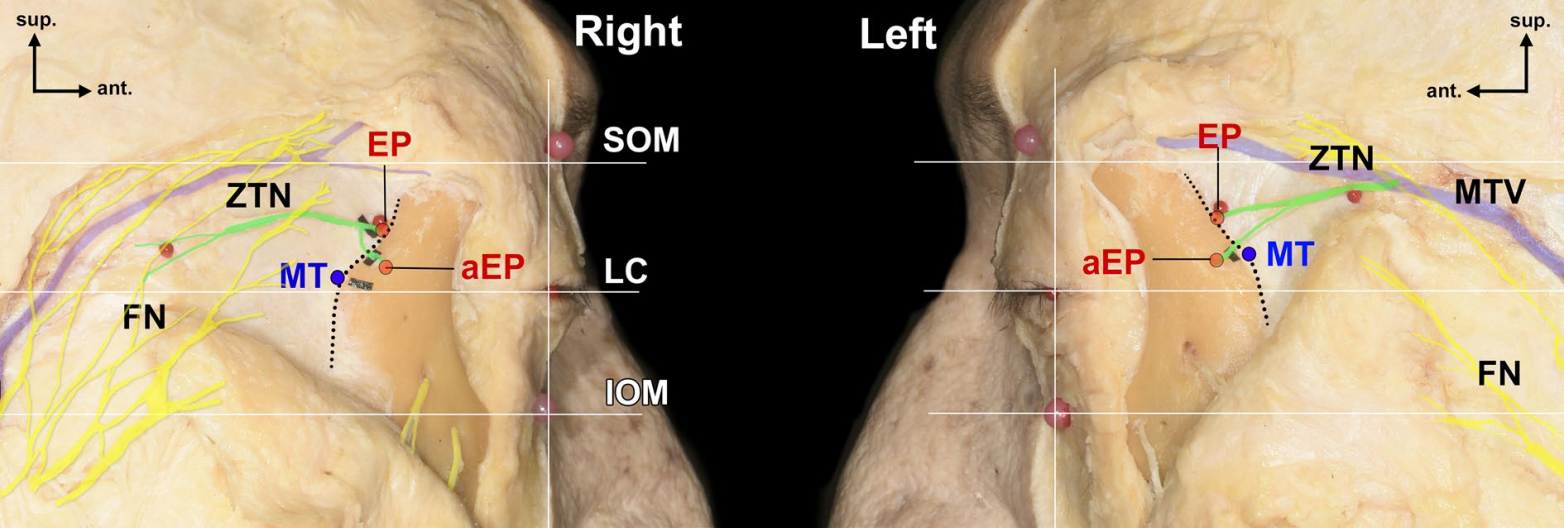
Bilateral but asymmetric distribution of the accessory zygomaticotemporal nerve. Cadaveric dissections of the right and left temporal regions demonstrating the zygomaticotemporal nerve (ZTN) and an accessory branch (aZTN). The main emergence point (EP) and accessory emergence point (aEP) are shown in relation to the marginal tubercle (MT). This figure illustrates a bilateral but asymmetric distribution of the aZTN. Facial nerve branches (FN) and the middle temporal vein (MTV) are shown for orientation.

The aEP was consistently identified near the MT. The distance from the MT to the aEP represented the primary outcome of this study, and the distance from the LC to the aEP was evaluated as the secondary outcome. Figure 2; Table 1 summarize all of the measured parameters. All measurements followed a normal distribution, and no statistically significant differences were found between sex or laterality. Among 36 hemifaces, the aZTN was identified in 7 out of 36 hemifaces (19.4%). It was unilateral in 5 hemifaces (3 cases observed on the right side and 2 on the left side) and bilateral in 2 hemifaces (Fig. 3).

**Table 1:** Measured parameters. Detailed descriptions are provided in the main text and Fig. 2.

Category	Anatomical parameter	#	Mean	SD
Location of aEP	Linear distance MT to aEP	(1)	3.4	1.2
	Horizontal distance from LC to aEP	(2)	22.3	2.4
	Vertical distance from LC to aEP	(3)	5.8	2.7
Location of MT	Horizontal distance from LC to MT	(4)	19.8	3.2
	Vertical distance from LC to MT	(5)	5.8	2.8
Facial dimension	Horizontal distance from LC to Tragion	(6)	86.1	4.9
	Horizontal distance from MOM to LOM	(7)	35.0	3.0
	Vertical distance from SOM to IOM	(8)	34.6	3.1

#, number of the measured parameter (Values are in millimeters).

aEP, accessory emergence point of the zygomaticotemporal nerve; MT, marginal tubercle; MOM, medial orbital margin; LOM, lateral orbital margin; SOM, supraorbital margin; IOM, infraorbital margin.

**Fig. 4:**
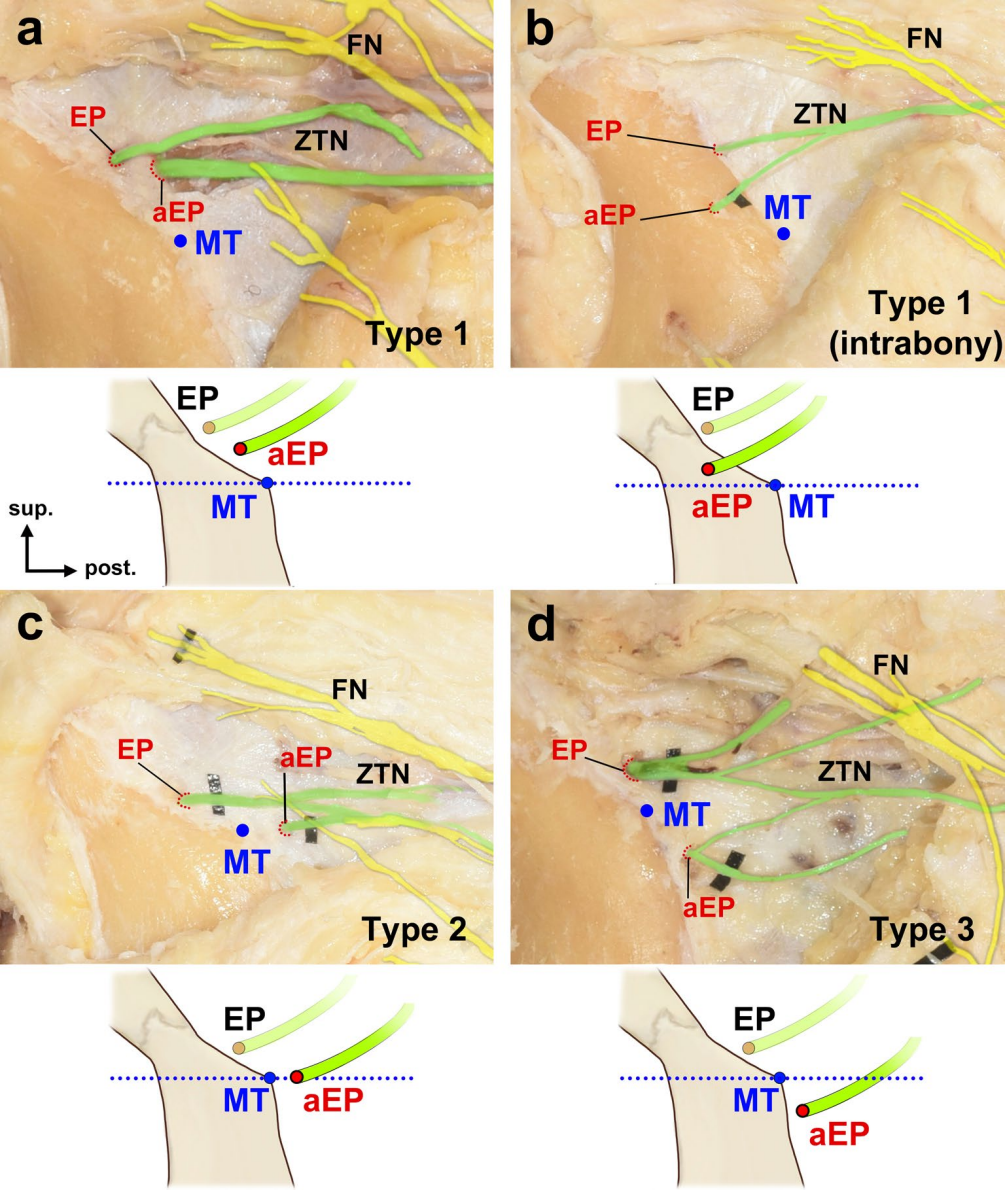
Classification of accessory emergence patterns relative to the marginal tubercle. Schematic composite illustration and representative cadaveric examples illustrating three types of accessory emergence points (aEP) of the accessory zygomaticotemporal nerve (aZTN) in relation to the marginal tubercle (MT). (**a**) Type 1, with the aEP located superior to the MT. (**b**) Type 1 (intrabony), in which the aZTN emerges through the zygomatic bone. (**c**) Type 2, with the aEP at the same horizontal level as the MT. (**d**) Type 3, with the aEP located inferior to the MT. The main emergence point (EP), zygomaticotemporal nerve (ZTN), and facial nerve branches (FN) are shown for orientation. Sup., superior; Post., posterior.

Based on the vertical relationship between the aEP and the MT, the aEPs were classified into three types: Type 1, superior to the MT; Type 2, at the same horizontal level as the MT; and Type 3, inferior to the MT (Fig. 4; Table 2). The type-specific linear distances from the MT to the aEP, as well as the corresponding horizontal and vertical offsets, are summarized in Table 2. Overall, the aEPs were located within a few millimeters of the MT, supporting the use of the MT as a central landmark for estimating the accessory branch emergence region.

**Table 2:** Type-specific location of the accessory emergence point relative to the marginal tubercle (MT).

Type 1 (Superior to MT)	Number	Linear distance from MT	Horizontal (Mediolateral) distance	Vertical (Superoinferior) distance	Estimated skin-to-sDTF depth
	4	3.6	− 1.8	2.2	6.10
Type 2 (On the horizontal line through the MT)	1	3.3	3.3	0.0	5.32
Type 3 (Inferior to MT)	2	2.9	2.5	− 1.8	6.38
Overall	7	3.4	0.1	0.7	5.92

Values are in millimeters.

sDTF, superficial layer of the deep temporal fascia; On the horizontal axis, positive values indicated positions temporal to the vertical line passing through the MT; On the vertical axis, positive values indicated positions superior to the horizontal line passing through the MT.

Type 1 (superior to the MT) was the most common pattern, observed in four of seven cases (57.1%), with the aEP located 3.0 ± 1.0 mm superior to the MT based on linear distance measurements. Type 2 (at the same level as the MT) was observed in one case (14.3%), with the aEP located 3.4 mm lateral to the MT, and Type 3 (inferior to the MT) was found in two cases (28.6%), with the aEP located 2.9 ± 1.7 mm inferior to the MT. In two Type 1 specimens, the aZTN showed a bone-emergent, intrabony variant. In these cases, the accessory branch appeared to exit through the frontal process of the zygomatic bone and reach the subcutaneous plane, rather than following the usual interfascial course between the sDTF and dDTF (Fig. 4B). The mean distance from the MT to the aEP across all specimens was 3.4 ± 1.4 mm, while in the two cases where the nerve emerged through the bone, the distance from the MT to the bony emergence point averaged 4.2 ± 0.1 mm.

Regarding the secondary outcome, the horizontal and vertical distances from the LC to the aEP were 22.3 ± 2.4 mm and 5.8 ± 2.7 mm, respectively (Fig. 2). The corresponding distances from the LC to the MT were 19.8 ± 3.2 mm horizontally and 5.8 ± 2.8 mm vertically. For reference, general facial measurements were also recorded, including the horizontal distance from the MOM to the LOM (35.0 ± 3.0 mm) and the vertical distance from the SOM to the IOM (34.6 ± 3.1 mm) (Fig. 2). In exploratory correlation analysis suggested a trend in which greater width (#7) was positively associated with an increased horizontal LC–aEP distance (#2) (*r* = 0.835), and that a larger orbital height (SOM–IOM distance [#8]) associated with a greater vertical LC–aEP distance (#3) (*r* = 0.972, *P* = 0.006). These findings suggest that the LC may serve as a complementary surface landmark for estimating the aEP. Because LC-based distances appeared to vary with facial dimensions, the LC may help account for interindividual differences in facial size.

**Fig. 5:**
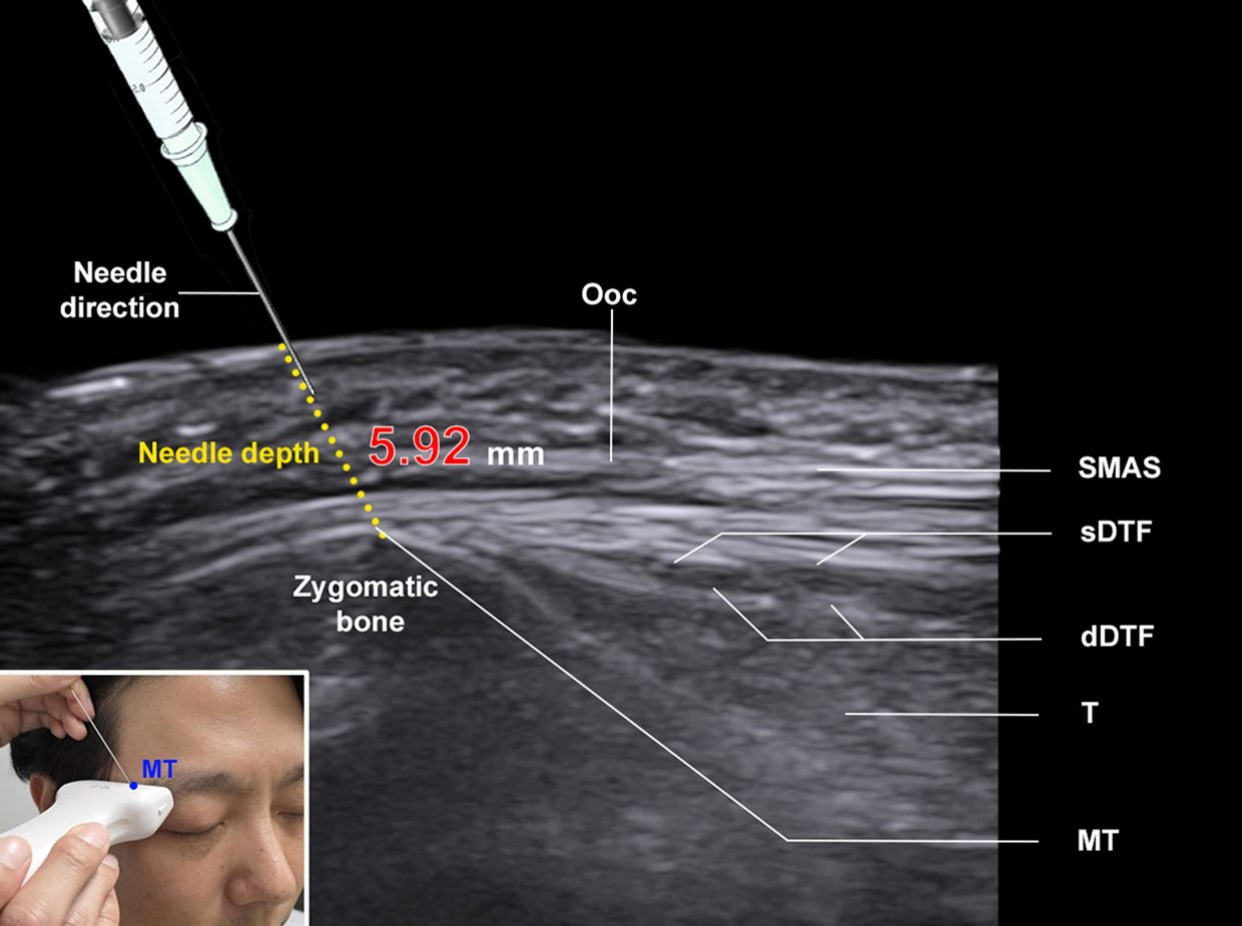
Ultrasonographic image of the temporal region showing the depth from the skin surface to the superficial layer of the deep temporal fascia (sDTF) near the MT. The aZTN was not directly visualized; the measured depth represents an in vivo estimate of the target fascial plane relevant to ZTN/aZTN blockade. Layered structures, including the SMAS, sDTF, deep layer of the deep temporal fascia (dDTF), temporalis muscle (T), orbicularis oculi muscle (Ooc), and zygomatic bone, are labeled. Inset shows probe positioning during in vivo examination.

In cross-sectional ultrasonographic observations, the depth from the skin surface to the superficial layer of the deep temporal fascia (sDTF) was measured near the MT in living subjects. The aZTN itself was not directly visualized by ultrasound; instead, the skin-to-sDTF depth was used as an in vivo reference for the target interfascial plane where the ZTN and potential accessory branches are expected to course based on cadaveric anatomy. The mean skin-to-sDTF depth at the MT region was 5.92 ± 0.99 mm (range, 3.92–8.51 mm) (Fig. 5). For type-specific depth estimates, skin-to-sDTF depth was measured at locations corresponding to the mean vertical positions of each aEP type relative to the MT: approximately 3 mm superior to the MT for Type 1, at the MT level for Type 2, and approximately 3 mm inferior to the MT for Type 3. The estimated skin-to-sDTF depths were 6.10 ± 0.90 mm for Type 1, 5.32 ± 0.88 mm for Type 2, and 6.38 ± 0.91 mm for Type 3 (Table 2).

## Discussion

This study aimed to clarify the emergence points of aZTN with reference to the MT, and to evaluate the reliability of the MT as an anatomical landmark. Based on the dissection of 36 hemifaces, the aZTN was identified in 7 cases (19.4%). The analysis of the aEPs relative to the MT demonstrated a mean distance of 3.4 ± 1.4 mm. The aEPs were classified into three patterns based on their vertical relationship to the MT: Type 1 (superior to MT) was the most common (57.1% of cases), located at a mean vertical distance of 2.2 mm; Type 3 (inferior to MT) was observed in 28.6% (mean vertical distance − 1.8 mm); and Type 2 (on the horizontal line through MT) in 14.3%. Utilizing the lateral canthus (LC) as an alternative landmark, the aEP was located 22.3 ± 2.4 mm horizontally and 5.8 ± 2.7 mm vertically from the LC. Furthermore, in vivo ultrasonography in healthy volunteers determined the mean depth from the skin surface to the sDTF, approximating the emergence depth, as 5.92 ± 0.99 mm at the MT region.

While numerous anatomical investigations have focused on the course of the main trunk of the ZTN, the ZTN exhibits considerable anatomical variation, including the frequent presence of accessory branches. The existence of these aZTN is clinically important because failure to account for them can lead to incomplete nerve blocks or anesthesia failure, and may also contribute to inter-individual differences in pain patterns during temporal filler or lifting procedures. In previous work and related studies, the MT was established as a reliable, palpable external landmark for accurately locating the EP of the main ZTN^11^; the present study further extends this clinical utility by quantifying the precise anatomical relationship between the MT and the aEP to improve the consistency and efficacy of regional nerve blocks.

Previous anatomical and clinical studies have demonstrated that the ZTN frequently shows branching or foraminal variation, including accessory or irregular temporal branches. These findings, summarized in Table 3, support the concept that accessory sensory pathways may contribute to incomplete anesthesia when only the conventional main ZTN location is targeted. The present study extends these observations by providing MT- and LC-referenced localization of the aEP and by adding ultrasound-based depth information relevant to the target fascial plane.

**Table 3:** Prevalence and frequency of accessory ZTN and related variations.

References	Study design	Anatomical variation evaluated	Sample size	Reported prevalence
Janis et al.^6^	Cadaveric dissection	Two ZTN branches; separate foramina transmitting branches	Specimens (not specified here)	Two ZTN branches in 30%; two separate foramina in 6%
Akita et al.^13^	Cadaveric dissection	Irregular branch through a small canal at the sphenozygomatic suture	42 sides	7/42 sides (16.7%)
Present study	Cadaveric dissection	Accessory ZTN (aZTN) prevalence	36 hemifaces	7/36 hemifaces (19.4%)
Totonchi et al.^7^	Clinical (live patients; surgical observation)	Additional accessory branch(es)	20 patients	≥ 1 accessory branch in 12/20 (60%); two accessory branches in 3/20 (15%)
Grimes et al.^9^	Imaging (micro-CT)	Multiple exterior openings of the ZTF	8 subjects	Two exterior openings in 5/8 (62.5%)

ZTF, zygomaticotemporal foramen.

Although the reported location of aZTN branches varies across studies, prior clinical mapping generally places these branches close to the main ZTN trunk—described as superior, posterolateral, or immediately adjacent^7,11^—rather than in a distant territory. Consistent with this concept, we identified an aZTN in 7/36 hemifaces (19.4%), and its emergence point (aEP) clustered around the MT region, with a mean MT–aEP distance of 3.4 mm. Although the aEP showed directional variability relative to the MT (Type 1: 57.1% at 3.0 mm superior; Type 3: 28.6% at 2.9 mm inferior; Type 2: 14.3% at the same level), the offsets remained within only a few millimeters of the MT, and MT-referenced measurements similarly supported a narrow vertical range. Taken together with our prior finding that the main ZTN emergence point lies 4.5 ± 1.6 mm superior to the MT^14^, these data suggest that aZTN emergence typically does not deviate far from the main ZTN “field,” supporting an MT-centered search/block strategy to capture both pathways.

In response to clinical guidance on injection depth, prior landmark-guided techniques for ZTN block have generally used broad, tactile endpoints to place local anesthetic in the correct fascial plane while reducing the risk of deeper injury. Totonchi et al.^7^ recommended advancing a 30-gauge, 2.5-cm needle vertically until distinct resistance is felt at about 1–2 cm (interpreted as fascial penetration), then injecting 0.5–1 mL just beneath the sDTF. This fascial distinction is clinically important because the frontal branch of the facial nerve lies more superficially near the STF, whereas the ZTN and aZTN are related to the deeper DTF layers. This plane is anatomically reasonable because the frontal branch of the facial nerve (FN) runs more superficially (between the STF and the sDTF)^15,16^, whereas the ZTN lies deeper, between the sDTF and the dDTF. Therefore, injections placed too superficially may inadvertently block the FN, while targeting just deep to the sDTF is more likely to anesthetize the ZTN while sparing the motor nerve^17^.

Whereas earlier guidance relied mainly on coarse depth ranges (e.g., “1–2 cm until resistance”), our study adds ultrasound-based quantitative depth information relevant to the aZTN. Using in vivo ultrasonography at the MT level, we measured the depth from the skin to the sDTF—used as the aZTN emergence-depth plane—as 5.92 ± 0.99 mm (range, 3.92–8.51 mm). This MT-centered, imaging-derived estimate can complement landmark methods by offering a more precise reference for placing anesthetic just deep to the sDTF.

**Fig. 6:**
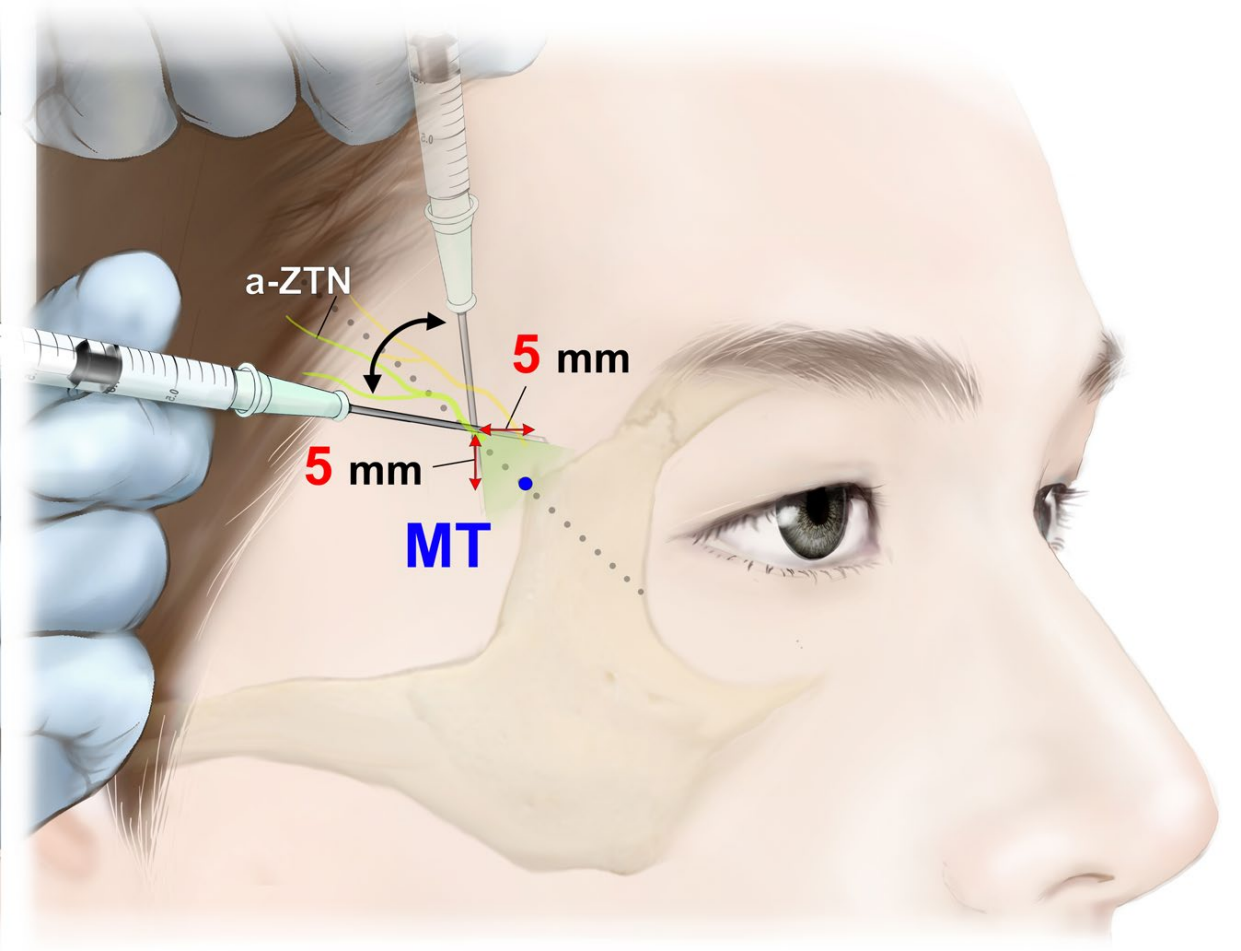
MT-centered two-point injection technique for temporal nerve block. Schematic illustration of a two-point injection strategy based on the marginal tubercle (MT). Injections are performed approximately 5 mm superior and 5 mm inferior to the MT, with the needle advanced perpendicular to the skin to the target depth. A limited fan-shaped redirection within the same depth plane may be used to improve coverage of the zygomaticotemporal nerve and its accessory branches.

Based on the above findings, an MT-centered two-point approach may be considered as an anatomically informed strategy for future evaluation, with the goal of improving regional nerve block reliability in the temporal region. (Fig. 6). Given that the main ZTN emerges approximately 4 mm superior to the MT^11^ while aZTN show vertical variability of ± 3 mm around the MT, a single-point approach may fail to cover accessory branches in some cases. In this conceptual approach, two injection points would be placed approximately 5 mm superior and 5 mm inferior to the MT, with the needle directed toward the plane immediately deep to the sDTF, guided by the observed skin-to-sDTF depth. This strategy may theoretically improve coverage of both the main trunk and accessory branches compared with a single-point approach. When broader coverage is needed, a fan-shaped dispersion could be considered as part of this conceptual approach by redirecting the needle laterally within the same depth plane without further advancement. Further validation through dye-spread studies, imaging confirmation of injectate distribution, and clinical outcome assessments is needed to determine whether this strategy can improve nerve block reliability and to establish optimal injection parameters for clinical use.

A further clinical nuance is the occasional bone-emergent, intrabony aZTN variant. In two Type 1 specimens, the aZTN appeared to exit through the frontal process of the zygomatic bone and reach the subcutaneous plane, rather than following the usual interfascial course between the sDTF and dDTF. This intrabony variant may be clinically relevant because an injection limited to the interfascial plane just deep to the sDTF may not fully cover a branch that exits directly through bone into a more superficial plane. Therefore, when residual temporal pain persists despite an otherwise appropriate MT-centered, sDTF-targeted block, clinicians may consider supplementary field infiltration around the MT or an additional LC-referenced approach. In our measurements using the LC as the reference point, the aEP was typically located 22.3 ± 2.4 mm lateral and 5.8 ± 2.7 mm superior to the LC, providing a complementary surface estimate for placing a small additional field of anesthetic to improve coverage of potential bone-piercing branches.

This study has several limitations. First, most cadaveric specimens were obtained from elderly individuals, and the location of the nerve may differ in younger adults and children because cranio-orbital anatomy changes with growth^18^. Second, the cadaveric specimens were East Asian, and orbital and temporal anatomical features may vary across racial and ethnic populations^19,20^; therefore, the applicability of MT- and LC-based landmark relationships to other populations should be interpreted with caution. Third, the ultrasonographic cohort consisted of young healthy adults, whereas the cadaveric specimens were elderly. Age-related differences in soft-tissue thickness, subcutaneous tissue distribution, and fascial anatomy may have affected the skin-to-sDTF depth measurements. Thus, the ultrasound data should be interpreted as an approximate in vivo reference rather than direct age-matched validation of the cadaveric findings. Fourth, we used embalmed cadavers rather than fresh cadavers or in vivo anatomical measurements. Although formalin fixation can reduce tissue dimensions by approximately 4%, this degree of shrinkage is modest and is unlikely to substantially alter the relationship of the aEP to fixed bony landmarks, such as the MT and LC^21,22^. Nevertheless, future studies using fresh cadavers, age-matched volunteers, and ethnically diverse clinical populations are needed to confirm these landmark relationships and depth estimates.

In conclusion, this anatomical study quantitatively localizes the aEP using the MT as a palpable external landmark and adds in vivo ultrasound-derived skin-to-sDTF depth information near the MT. Together with prior MT-based mapping of the main ZTN, these findings provide an anatomically grounded framework for future evaluation of MT-centered strategies targeting the plane immediately deep to the sDTF, including two-point or limited fan-shaped approaches to address accessory ZTN branches. Further dye-spread, imaging-guided, and clinical outcome studies are needed before specific injection parameters can be recommended for routine clinical practice.

## Data Availability

The datasets generated and analyzed during the current study are available from thecorresponding author on reasonable request.
